# Trotter and Gleser’s (1958) equations outperform Trotter and Gleser’s (1952) equations in stature estimation of the US White males

**DOI:** 10.1093/fsr/owad008

**Published:** 2023-04-29

**Authors:** Yangseung Jeong, Rebecca J Taylor, Yochun Jung, Eun Jin Woo

**Affiliations:** Department of Biology, Middle Tennessee State University, Murfreesboro, USA; Defense POW/MIA Accounting Agency-Laboratory, Joint Base Pearl Harbor-Hickam, Hickam, USA; Department of Thoracic and Cardiovascular Surgery, Chonnam National University Hospital, Gwangju, Republic of Korea; Department of History, Sejong University, Seoul, Republic of Korea

**Keywords:** forensic anthropology, stature estimation, Trotter and Gleser, FORDISC, White males, Bayes factor

## Abstract

Trotter and Gleser presented two sets of stature estimation equations for the US White males in their 1952 and 1958 studies. Following Trotter’s suggestion favouring the 1952 equations simply due to the smaller standard errors, the 1958 equations have been seldom used and have gone without additional systematic validation tests. This study aims to assess the performance of the Trotter and Gleser 1952, Trotter and Gleser 1958, and FORDISC equations for the White males in a quantitative and systematic way, particularly when applied to the WWII and Korean War casualties. In sum, 27 equations (7 from the 1952 study, 10 from the 1958 study, and 10 from FORDISC) were applied to the osteometric data of 240 accounted-for White male casualties of the WWII and Korean War. Then, the bias, accuracy, and Bayes factor for each set of stature estimates were calculated. The results show that, overall, Trotter and Gleser’s 1958 equations outperform the 1952 and FORDISC equations in terms of all three measures. Particularly, the equations with higher Bayes factors produced stature estimates where distributions were closer to that of the reported statures than those with lower Bayes factors. When considering Bayes factors, the best performing equation was the “Radius” equation from the 1958 study (BF = 15.34) followed by the “Humerus+Radius” equation from FORDISC (BF = 14.42) and the “Fibula” equation from the 1958 study (BF = 13.82). The results of this study will provide researchers and practitioners applying the Trotter and Gleser stature estimation method with a practical guide for equation selection.

**Key Points:**

- The performance of three stature estimation methods was compared quantitatively.
- Trotter and Gleser’s (1952, 1958) and FORDISC White male equations were included.
- Overall, Trotter and Gleser’s 1958 method outperformed the other methods.
- This study provides a practical guide for stature estimation equation selection.

## Introduction

Stature has been extensively studied in various fields of anthropology as an important biological property indicative of the health, environmental conditions, and even socio-economic/political circumstances of an individual and population [1–7]. As direct measurement of an individual’s stature is not always feasible, particularly when they are deceased, extensive effort has been made to devise methods to estimate stature from skeletal elements since the late 19th century [8–15]. Since its introduction in the 1950s, Trotter and Gleser’s [14, 15] method has become one of the most popular techniques for stature estimation [16–18].

“Trotter and Gleser’s method” refers to a set of stature estimation equations devised from their studies published in 1952 and 1958. In the 1952 study, the authors presented equations for the White and Black individuals using the World War II (WWII) US service member casualties and Terry Collection samples [14]. In 1958, they provided male equations for the Whites, Blacks, Asians, Puerto Ricans, and Mexicans using US Korean War service member casualties [15]. Trotter [19] explicitly suggested using the 1952 equations for White and Black males over the 1958 equations due to the smaller standard errors in the 1952 equations compared with the 1958 equations. Following Trotter’s [19] suggestion, Trotter and Gleser’s 1958 equations have been seldom applied to White and Black males and have gone without additional systematic validation tests. In fact, the stature estimation tool built into FORDISC, a forensic anthropological analysis software popular among forensic practitioners, is based on the Trotter and Gleser’s 1952 dataset, not their 1958 dataset, to estimate statures of the 20th century (20C) Whites and Blacks [20]. However, a standard error associated with a certain regression equation is not necessarily a measure of its performance as it is not a predictive estimator but simply a descriptive indicator of the overall discrepancy between the actual and estimated values in a dataset used for the equation development [17, 21].

Besides Trotter’s [19] suggestion of using standard error, a general lack of feasible quantitative methods to compare the performance of different stature estimation methods explains the lack of systematic validation tests of Trotter and Gleser’s 1952 and 1958 equations. Comparing the mean of stature estimates obtained by an estimation method to that of known statures has often been used for comparing the relative performance of a method [22, 23]. However, as Jeong and colleagues [24] highlight, even identical means do not necessarily indicate that the estimates and known statures follow a same-shaped distribution and thus, cannot guarantee a good performance of the method. In this regard, Jeong et al. [24] suggest that, given the distributions of known statures and estimated statures, Bayes factors can be used to compare the performance of multiple stature estimation methods quantitatively and objectively.

Bayes factor refers to a ratio of the marginal likelihoods for two models [25, 26]. In the context of stature estimation, the two models will be the distributions of two sets of stature data (e.g. estimated statures and known statures). The distribution of multiple sets of estimated statures produced by different estimation methods may be compared with the same distribution (i.e. distribution of known statures) so that the relative performance of the methods can be assessed using Bayes factors [24]. In other words, a method with a greater Bayes factor can be concluded to perform better than that of a lower Bayes factor.

The goal of this study is to assess the performance of Trotter and Gleser’s 1952 and 1958 White male equations using Bayes factors when applied to the WWII and Korean War casualties. Black males were excluded from this study due to a small sample size. Bayes factors for each equation will be calculated by comparing the distribution of the stature estimates to that of the reported living statures. Thus, the resultant Bayes factors will indicate the relative performance of the equations, which will help researchers select the best equation available to them. To the authors’ knowledge, this is the first effort to validate Trotter and Gleser’s 1952 and 1958 equations in a systematic and quantitative way. The result of this study is expected to be beneficial to any researchers who estimate statures of skeletal remains using the Trotter and Gleser’s method, particularly those who work on the identification of the WWII and Korean War casualties.

## Materials and methods

### Data

The living stature and long bone measurement data were obtained from 240 accounted-for White male casualties of the WWII and Korean War whose skeletal remains were accessioned into the Defense POW/MIA Accounting Agency Laboratory (DPAA-Lab) and/or its predecessor organisations (JPAC and CILHI) between 1989 and 2017. Every individual used in this study had documented living statures from their antemortem records and possessed at least one of the measurable long bones from their upper and/or lower limbs. Living statures originally recorded in inches were transformed into centimeters by multiplying 2.54 and then rounding the values up to one decimal place. All bone measurements were taken by certified forensic anthropologists at the DPAA-Lab using the contemporary standards [27–29]. When both sides of bones were present, stature estimates were produced using left bones, and right bones were used only when their left counterparts were unavailable.

### Equations to be compared

In their 1952 study, Trotter and Gleser presented seven simple regression equations and three multiple regression equations for White males [14, p.495]. In 1958, they presented another set of 10 equations, and all are simple regression equations [15, p.120]. In Trotter’s [19] study, only the seven simple regression equations from the 1952 study were included in her recommendations to “give satisfactory estimates”. Maximum lengths of six individual limb bones (humerus, radius, ulna, femur, tibia, and fibula) as well as the summed length of the “Femur+Tibia” were used to develop the simple regression equations in both 1952 and 1958 studies and, thus, these seven equations were compared in this study. Although three simple regression equations in the 1958 study using the summed lengths of “Femur+Fibula”, “Humerus+Radius”, and “Humerus+Ulna” were not presented in the 1952 study, they were included for comparison in this study because FORDISC, which is based on Trotter and Gleser’s 1952 data, provides stature estimates using those summed lengths. Three multiple regression equations from the 1952 study could not be compared with any of the 1958 equations and thus, were excluded from this study (Table 1). Even though FORDISC is based on Trotter and Gleser’s [14] WWII data, it uses slightly different equations from those of Trotter and Gleser’s 1952 study. Thus, with the “20th MStat” and “WM” options selected, the performance of FORDISC-generated stature equations was also compared with the Trotter and Gleser’s 1952 and 1958 equations. A total of 27 sets of stature estimates were produced for comparison: 7 using the 1952 equations, 10 using the 1958 equations, and 10 using FORDISC (Table 1).

**Table 1:** Stature estimation equations in Trotter and Gleser’s 1952 and 1958 studies and FORDISC.

Equations	T&G’52 [14]	T&G’58 [15]	FORDISC (version 3)^c^
Simple regression equation^a^	3.08 × Hum+70.45 3.78 × Rad+79.01 3.70 × Ulna+74.05 2.38 × Fem + 61.41 2.52 × Tib + 78.62 2.68 × Fib+71.78 1.30 × (Fem + Tib) + 63.29	2.89 × Hum+78.10 3.79 × Rad+79.42 3.76 × Ulna+75.55 2.32 × Fem + 65.53 2.42 × Tib + 81.93 2.60 × Fib+75.50 1.26 × (Fem + Tib) + 67.09 1.31 × (Fem + Fib) + 63.05 1.82 × (Hum+Rad) + 67.97 1.78 × (Hum+Ulna) + 66.98	2.7615 × Hum+81.36 3.5654 × Rad+84.8 3.4760 × Ulna+80.56 2.2285 × Fem + 68.64 2.3587 × Tib + 82.41 2.5163 × Fib+78.00 1.2244 × (Fem + Tib) + 68.56 1.2845 × (Fem + Fib) + 64.29 1.7195 × (Hum+Rad) + 73.30 1.7347 × (Hum+Ulna) + 69.19
Multiple regression equation^b^	1.42 × Fem + 1.24 × Tib +59.88 0.93 × Hum+1.94 × Tib + 69.30 0.27 × Hum+1.32 × Fem + 1.16 × Tib + 58.57		

Hum: humerus; Rad: radius; Uln: ulna; Fem: femur; Tib: tibia; Fib: fibula.

a Equations included in this study for comparison. Note that these are the equations included in Trotter [19].

b Equations excluded from this study due to a lack of Trotter and Gleser’s 1958 equations to be compared.

c Only the equations used for comparison in this study are listed.

It should be noted that the tibial measurements in FORDISC have been adjusted by the developers [20] due to the possible error pointed out by Jantz and colleagues [30, 31]. However, when Trotter and Gleser’s 1952 and 1958 tibia equations were applied in this study, the maximum tibial lengths (i.e. condylo-malleolar length) were entered into the equations with no corrections/adjustment. For the rest of analyses, a point estimate was regarded as the estimated stature of an individual.

### Performance comparison among equations

Bayes factors along with associated posterior probabilities were calculated to compare the performance of the equations. Additionally, two frequently used performance measures were calculated for comparability purposes: bias (i.e. mean of differences between the estimated and actual statures) and accuracy (i.e. mean of absolute differences between the estimated and actual statures). Bayes factor calculation requires to specify the type of data distributions, so Kolmogorov–Smirnov tests were conducted to test for normality for the 27 sets of stature estimates and reported statures (i.e. documented living statures). Additionally, histograms, kurtosis, and skewness were drawn/calculated to confirm that there is no significant departure of the data from a normal distribution. All analyses and visualisation of data were conducted using RStudio version 1.3.959 for Windows [32]. The LearnBayes package and R code provided in Jeong et al. [24] were used to calculate Bayes factors and posterior probabilities.

## Results

Of 240 individuals, the numbers of individuals having measurable humerus, radius, ulna, femur, tibia, and fibula were 182, 156, 139, 200, 191, and 155, respectively (Table 2). Approximately 40%–60% of the individuals possessed both the left and right bones with only 11.5%–23.2% of individuals having just right bones (Table 2).

**Table 2:** Number of individuals having measurable limb bones.

Items	Left only (*n* (%))^a^	Right only (*n* (%))^a^	Both sides (*n* (%))^a^	Total
Hum	70 (38.5)	24 (13.2)	88 (48.4)	182
Rad	64 (41.0)	28 (17.9)	64 (41.0)	156
Uln	49 (35.3)	30 (21.6)	60 (43.2)	139
Fem	53 (26.5)	23 (11.5)	124 (62.0)	200
Tib	64 (33.5)	29 (15.2)	98 (51.3)	191
Fib	52 (33.5)	36 (23.2)	67 (43.2)	155

Hum: humerus; Rad: radius; Uln: ulna; Fem: femur; Tib: tibia; Fib: fibula.

^a^Percentages may not sum to 100 due to rounding.

Table 3 presents the descriptive statistics of the reported statures as well as the maximum lengths of the left and right bones. The mean stature (174.8 cm) in the current study is slightly greater than those reported in Trotter and Gleser’s 1952 and 1958 studies (174.0 cm and 173.95 cm, respectively); however, the difference was not statistically significant (one sample *t*-test; *P* > 0.05). The discrepancies in the mean bone lengths between the current study and the previous studies were as small as 0.02–0.85 cm with no statistical significance (one sample *t*-test; *P* > 0.05 for all bones) (Table 3).

**Table 3:** Descriptive statistics of the reported statures and maximum bone lengths (in cm) in comparison with Trotter and Gleser’s 1952 and 1958 data.

Items	Current study					T&G’52 [14]			T&G’58 [15]	
	*n*	Mean	SD	Min–Max		Mean	SD		Mean	SD
Reported stature	240	174.8	6.49	160.7–191.8		174.000	–		173.950	–
Hum left	158	33.7	1.76	29.7–38.2		33.595	1.672		33.562	1.663
Hum right	112	33.5	1.90	29.6–38.2		33.640	1.691		33.641	1.708
Rad left	128	25.1	1.37	21.8–28.1		25.058	1.271		25.147	1.277
Rad right	92	25.2	1.40	22.3–28.5		25.243	1.338		25.306	1.274
Uln left	109	26.9	1.38	24.1–29.5		26.938	1.285		27.005	1.303
Uln_right	90	27.0	1.45	24.3–30.8		27.131	1.302		27.174	1.321
Fem left	177	47.4	2.51	41.4–53.7		47.290	2.357		47.150	2.345
Fem right	147	47.0	2.53	41.6–53.2		47.232	2.358		47.077	2.382
Tib left^a^	162	38.7	2.37	33.5–45.1		37.854	2.187		38.457	2.214
Tib right^a^	127	38.3	2.33	33.2–43.7		37.799	2.186		38.429	2.226
Fib left	119	38.3	2.33	32.6–44.8		38.153	2.107		38.276	2.058
Fib right	103	38.1	2.24	32.9–43.4		38.118	2.074		38.258	2.084

Hum: humerus; Rad: radius; Uln: ulna; Fem: femur; Tib: tibia; Fib: fibula.

a Condylo-malleolar length of the tibia.

**Figure 1:**
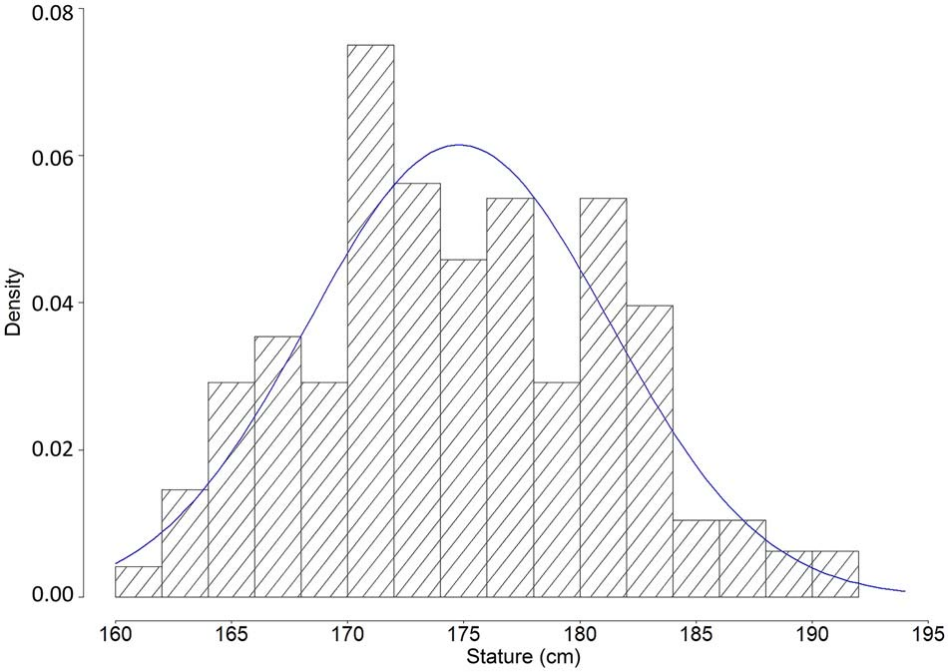
Histogram of reported stature data with a normal distribution curve (kurtosis = 2.469; skewness = 0.197).

The results of the Kolmogorov–Smirnov tests indicate that all the 27 sets of stature estimates are normally distributed (*P* > 0.05) (Table 4). The reported statures yield a *P*-value of 0.05 with a D statistic of 0.089 (Table 4). This relatively low *P*-value is likely due to a large sample size (*n* = 240), which makes kurtosis somewhat sensible [33]. The kurtosis of 2.469 implies a slightly platykurtic distribution of the data with relatively heavy tails; however, the histogram shows that the data do not depart from a normal distribution significantly (Figure 1). Also, the skewness of 0.197 is close enough to zero indicating that the data are not positively or negatively skewed [33]. Thus, it was concluded that all sets of estimated statures and reported statures follow a normal distribution.

**Table 4:** Kolmogorov–Smirnov test results for 27 sets of stature estimates and reported statures.

Study	Equation	D statistic	*P*
T&G’52	Hum	0.051417	0.72
	Rad	0.058382	0.66
	Uln	0.069841	0.51
	Fem	0.038037	0.93
	Tib	0.044290	0.85
	Fib	0.053124	0.77
	Fem + Tib	0.060753	0.55
T&G’58	Hum	0.051422	0.72
	Rad	0.057951	0.67
	Uln	0.069657	0.51
	Fem	0.037996	0.94
	Tib	0.044280	0.85
	Fib	0.053382	0.77
	Fem + Tib	0.060896	0.55
	Fem + Fib	0.049934	0.88
	Hum+Rad	0.071617	0.51
	Hum+Uln	0.062647	0.74
FORDISC	Hum	0.051350	0.72
	Rad	0.057901	0.67
	Uln	0.069614	0.51
	Fem	0.037966	0.94
	Tib	0.044185	0.85
	Fib	0.053642	0.76
	Fem + Tib	0.060630	0.56
	Fem + Fib	0.049623	0.88
	Hum+Rad	0.071615	0.51
	Hum+Uln	0.062324	0.74
Reported stature	–	0.088811	0.05

Hum: humerus; Rad: radius; Uln: ulna; Fem: femur; Tib: tibia; Fib: fibula.

Table 5 reports the bias, accuracy, Bayes factors, and posterior probabilities associated with the stature estimates produced by the 27 equations. The bias (i.e. ∑(estimated stature − actual stature)/*n*) ranged −1.23 to 1.09 cm, −0.49 to 1.89 cm, and − 1.34 to (−0.52) cm for the Trotter and Gleser 1952, Trotter and Gleser 1958, and FORDISC equations, respectively. Overall, the 1952 and FORDISC equations tended to underestimate statures. Except for the “Tibia” and “Femur+Tibia” equations in the 1952 study, all the 1952 and FORDISC equations yielded a bias of −0.5 cm or larger. No obvious tendency of over- or underestimation of stature was noticed among the 1958 equations, and the largest bias (1.89 cm) was found in the “Ulna” equation. When the three methods were compared, the 1958 equations tend to yield the least bias except for the “Ulna” and “Femur+Tibia” equations, where the smallest values were obtained from the FORDISC (−0.76 cm) and 1952 equation (0.22 cm), respectively (Table 5).

**Table 5:** Performance comparison of Trotter and Gleser’s 1952 and 1958 and FORDISC stature equations.

Equation	Bias (cm)^a^			Accuracy (cm)^b^			Bayes factor			Posterior probability		
	T&G’52	T&G’58	FORDISC	T&G’52	T&G’58	FORDISC	T&G’52	T&G’58	FORDISC	T&G’52	T&G’58	FORDISC
Hum	−1.05	0.22^c^	−0.84	3.43	3.30^d^	3.44	1.66	9.89^e^	3.52	0.62	0.91	0.78
Rad	−1.15	−0.49^c^	−0.76	3.42	3.36^d^	3.40	3.46	15.34^e^	11.57	0.78	0.94	0.92
Uln	−1.23	1.89	−0.76^c^	3.54	3.98	3.52^d^	1.31	<0.01	7.47^e^	0.57	<0.01	0.88
Fem	−0.71	0.57^c^	−0.65	2.77	2.72^d^	2.75	2.27	8.75^e^	2.58	0.69	0.90	0.72
Tib	1.09	0.54^c^	−1.34	2.98	2.85^d^	2.98	0.57	6.33^e^	0.08	0.36	0.86	0.07
Fib	−0.53	0.14^c^	−0.56	2.84	2.76^d^	2.81	6.09	13.82^e^	5.24	0.86	0.93	0.84
Fem + Tib	0.22^c^	0.58	−1.00	2.58^d^	2.59	2.74	13.26^e^	8.29	0.70	0.93	0.89	0.41
Fem + Fib	–	0.42^c^	−0.52	–	2.49^d^	2.53	–	10.72^e^	5.68	–	0.91	0.85
Hum+ Rad	–	−0.39^c^	−0.98	–	3.22^d^	3.36	–	7.56	14.42^e^	–	0.88	0.94
Hum+Uln	–	−0.40^c^	−0.94	–	3.15^d^	3.21	–	12.30^e^	9.59	–	0.92	0.91

Hum: humerus; Rad: radius; Uln: ulna; Fem: femur; Tib: tibia; Fib: fibula.

a ∑(estimated stature − actual stature)/*n*.

b ∑|estimated stature − actual stature|/*n*.

c Equation(s) with the least bias (i.e. smallest value) among three methods under comparison.

d Equation(s) with the greatest accuracy (i.e. smallest value) among three methods under comparison.

e Equation(s) with the greatest Bayes factor among three methods under comparison.

A similar pattern was observed in terms of the accuracy (i.e. ∑|estimated stature − actual stature|/*n*). Compared with the other methods, the 1958 equations yielded better or similar accuracies except for the “Ulna” equation, where the FORDISC equation yielded the lowest value (3.52 cm). Overall, prioritising the three methods solely based on the accuracy did not appear practical because they tended to produce very similar values (e.g. “Femur+Tibia” equation) (Table 5).

Table 5 also shows that the 1958 equations produced the greatest Bayes factors among the three methods except for three equations (“Ulna”, “Humerus+Radius”, and “Femur+Tibia” equations). The greatest Bayes factors for the “Ulna” and “Humerus+Radius” equations were obtained from FORDISC (BF = 7.47 and 14.42, respectively) and the 1952 study yielded the greatest Bayes factor for the “Femur+Tibia” equation (BF = 13.26). Out of 27, 7 equations yielded Bayes factors greater than 10 indicating “strong evidence” of the scenario that the stature estimates come from the distribution of the population (i.e. known stature) [24, 40]. The seven equations with Bayes factors greater than 10 were the 1958 “Radius” equation (BF = 15.34), the FORDISC “Humerus+Radius” equation (BF = 14.42), the 1958 “Fibula” equation (BF = 13.82), the 1952 “Femur+Tibia” equation (BF = 13.26), the 1958 “Humerus+Ulna” equation (BF = 12.30), the FORDISC “Radius” equation (BF = 11.57), and the 1958 “Femur+Fibula” equation (BF = 10.72). It should be noted that four out of these seven equations are from Trotter and Gleser’s 1958 study (Table 5).

Posterior probabilities reported in Table 5 indicate how much the equations could improve their predictions compared with the prior conditions. As the prior probabilities were originally set as 0.5, posterior probabilities greater than 0.5 can be understood as an improvement of the equation’s performance. As indicated in Table 5, the equations with higher Bayes factors yielded higher posterior probabilities (Table 5).

**Figure 2:**
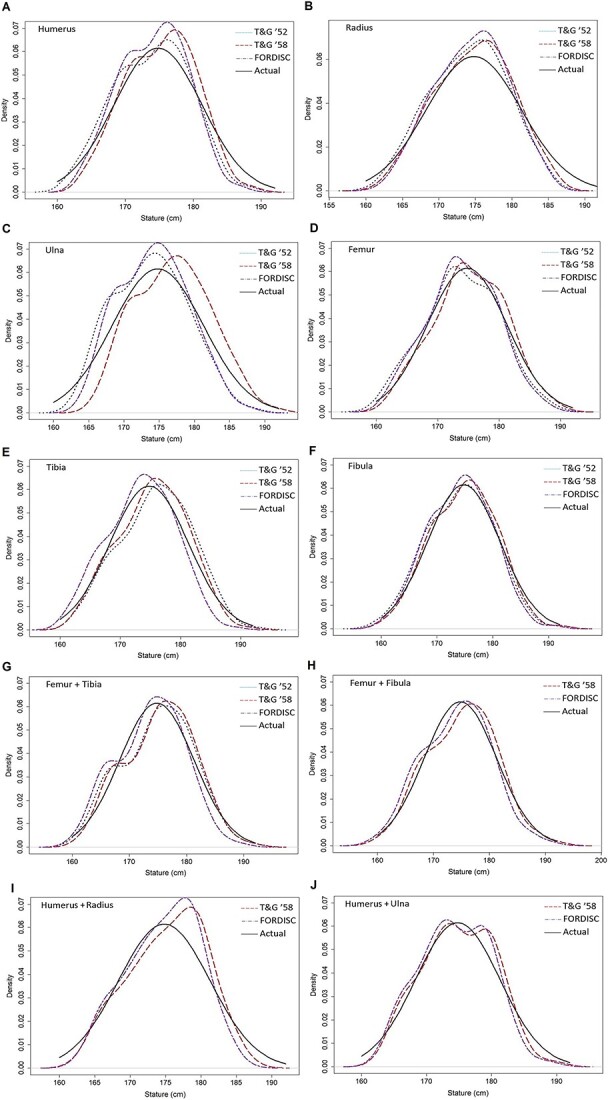
Graphical comparisons of the reported statures and estimated statures. The distributions of reported statures are represented by black solid lines and those of estimated statures by blue dotted line (Trotter and Gleser’s 1952 equations), red dash lines (Trotter and Gleser’s 1958 equations), and purple dot–dash lines (FORDISC equations), respectively.

Figure 2 presents graphical comparisons of the estimation methods by overlapping the distributions of the stature estimates with that of the reported statures. Overall, it was visually demonstrated that the equations with high Bayes factors tended to yield distributions of stature estimates which are similar to that of the reported statures.

## Discussion

Newly devised methods to reconstruct biological profile parameters (e.g. ancestry, sex, age-at-death, and stature) are generally expected to be subjected to vigorous validation processes by peer researchers using different samples and/or methodologies. For example, in a validation study of Pearson’s [9] stature estimation equations, Stevenson [34] found that accuracy might vary between populations and stressed the importance of a population-specific method for stature estimation. Also, multiple validation studies [35, 36] on Fully’s [11] technique led to a new version of the anatomical method using revised osteometric measurements and statistical methods by Raxter and colleagues [13]. As such, validation studies not only enhance the accuracy and applicability of the method but also serve as a basis for a new method development.

In general, to select a stature estimation method for a target sample, the similarities of biological (e.g. ancestry and sex), geographical, and temporal backgrounds between the target sample and the reference sample used to devise a method are regarded as important standards to be considered [19, 37]. Yet, no clear rule of thumb has been established for a situation where multiple methods meeting these standards are available for a target sample such as Trotter and Gleser’s White and Black male equations from their 1952 and 1958 studies. Although Trotter [19] favoured the 1952 equations, her suggestion was not based on an independent validation test but simply based on the comparison of the standard errors associated with each equation. As mentioned previously, as standard errors are not a predictive indicator, they should not be regarded as a proper measure to compare the performance of stature estimation equations. In this regard, Jeong and colleagues [24] suggest that (i) a good estimation method should yield stature estimates where distribution is similar to that of a population (i.e. known stature) and (ii) the similarity of the two distributions (i.e. distributions of estimated and known statures) can be assessed quantitatively using the Bayes factors.

Bayes factors, based on the Bayesian approach, have some practical advantages over a *P*-value obtained from hypothesis testing in a frequentist approach. First, unlike the *P*-value, which is used to determine if a null hypothesis can be simply rejected or not, the Bayes factor (BF_01_) presents the odds of how much more likely a set of given data would occur in the null model (M_0_) over the alternative model (M_1_). For example, given BF_01_ = 2, the Bayes factor suggests that (i) the given data are twice as likely to occur in the scenario of M_0_ compared with M_1_ and, at the same time, (ii) the given data are 0.5 times (i.e. 1/BF_01_) more likely to occur in the scenario of M_1_ compared with M_0_ [24, 38]. Moreover, the Bayes factors calculated from different datasets can be directly compared with each other, which is not possible for a *P*-value [38]. This study could compare the Bayes factors from 27 sets of stature estimates and reported statures due to this property of the Bayes factors, and assist in eventually prioritising the performance of the equations.

The Bayes factors were calculated in this study in a way that the distribution parameters (mean and standard deviation) of the reported statures and estimated statures were used for the null (M_0_) and alternative models (M_1_), respectively. Thus, the higher the Bayes factor (BF_01_), the more likely the scenario that the set of stature estimates occurs from the distribution of the reported statures (i.e. greater similarity between the distributions of the reported and estimated statures). Table 6 presents general guidelines to interpret the Bayes factors established by previous studies [39, 40]. In both Jeffreys’s [39] and Raftery’s [40] guidelines, a Bayes factor of 3 is regarded as a meaningful point beyond which can be interpreted as a “substantial” or “positive” evidence for the null model (Table 6). Furthermore, Jeffreys [39] specifies that the Bayes factor greater than 10 can be interpreted as “strong” evidence.

When considering Bayes factors, Trotter and Gleser’s 1958 study has more equations of greater performance than the other methods under comparison. About a half of the equations yielding Bayes factors greater than 3 (nine out of 19 equations) were from the 1958 study (Table 7). In fact, all 1958 equations except for the “Ulna” equation yielded the Bayes factors greater than 3. Moreover, more than half of the Bayes factors greater than 10 were obtained from the 1958 study (four out of seven) (Table 7). The greatest Bayes factor in this study was also obtained from one of the 1958 equations (i.e. the “Radius” equation yielding BF = 15.34) (Table 5). In addition, except for the “Ulna” and “Femur+Tibia” equations, the least bias and greatest accuracy were obtained from the 1958 equations. Overall, all these results indicate the 1958 equations outperform the 1952 and FORDISC equations.

This study provides researchers and practitioners applying the Trotter and Gleser stature estimation method with a practical guide for equation selection. In other words, based on the results presented in Tables 5 and 7, it is recommended to use the equation with the highest Bayes factor among the available options. It should be noted that the equation with the best Bayes factor is not necessarily associated with the lowest bias or greatest accuracy. For example, there are many equations with a lower bias and/or greater accuracy score than the 1958 “Radius” equation that yielded the greatest Bayes factor (BF = 15.34). Rather, the equation of a higher Bayes factor should be understood to produce stature estimates where distribution would mimic that of the true statures more accurately and thus, its overall performance would be greater than those with lower Bayes factors.

**Table 6:** Guidelines for interpretation of the Bayes factors suggested by Jeffreys [39] and Raftery [40]^a^.

Bayes factor (BF_01_)	Interpretation	
	Jeffreys [39]	Raftery [40]
10–20	Strong evidence for M_0_	Positive evidence for M_0_
3–10	Substantial evidence for M_0_	Positive evidence for M_0_
1–3	Anecdotal evidence for M_0_	Weak evidence for M_0_
1	No evidence	No evidence
1/3–1	Anecdotal evidence for M_1_	Weak evidence for M_1_
1/10–1/3	Substantial evidence for M_1_	Positive evidence for M_1_

a Partially retrieved from Jeong and colleagues [24].

**Table 7:** Classification of the equations under comparison by Bayes factors.

Method	Bayes factor			
	<1	1–3	3–10	>10
T&G’52	Tib	Hum Uln Fem	Rad Fib	Fem + Tib
T&G’58	Uln		Hum Fem Tib Fem + Tib Hum+Rad	Rad Fib Fem + Fib Hum+Uln
FORDISC	Tib Fem + Tib	Fem	Hum Uln Fib Fem + Fib Hum+Uln	Rad Hum+Rad

Hum: humerus; Rad: radius; Uln: ulna; Fem: femur; Tib: tibia; Fib: fibula.

As Jantz and colleagues [30, 31] raised the issue of possible mismeasurement of the tibia in Trotter and Gleser’s 1952 study, the accuracy of the tibia-related equations has been debatable [17, 41]. Jantz and colleagues [30, 31] speculated that, unlike the description presented in Trotter and Gleser [14], Trotter measured the maximum length of the tibia excluding the malleolus resulting in the overestimation of statures when the malleolus-included tibial length is plugged into the tibia equation. Jantz and Ousley [20] applied a correction factor to Trotter and Gleser’s [14] raw tibia measurement data and generated new tibia equations, which is currently built into FORDISC version 3, to address this issue. A 10-mm correction factor, which should compensate for the missing malleolus length, was intended to be applied; however, the correction factor was applied twice for an unknown reason and thus, the current tibia equation in FORDISC underestimates stature [41]. Trotter’s possible mismeasurement of the tibia in the 1958 study and the overcorrection of the tibial length in FORDISC explains the positive bias in the 1952 “Tibia” equation (1.09 mm) and negative bias in the FORDISC “Tibia” equation (−1.34 mm) as well as their low Bayes factors (BF = 0.57 and 0.08, respectively) (Table 5, Figure 2). On the other hand, the 1958 “Tibia” equation yielded a decent bias (0.54 mm) and Bayes factor (BF = 6.33). This result not only demonstrates the outperformance of the 1958 “Tibia” equation compared with the other methods but also supports the argument that there was no measurement issue with the tibia because the bones had not been measured by Trotter but the technicians following Trotter’s descriptions in the 1952 study.

Another somewhat unexpected finding from this study is the best Bayes factor was obtained from an upper limb equation (the 1958 “Radius” equation, BF = 15.34), as it is generally accepted that lower limb equations yield more accurate estimates compared with upper limb equations [16, 37]. The result of high-performing upper limb equations does not appear misguided considering that the Bayes factors greater than three were obtained more from the upper limb equations (10 out of 13 equations (77%)) than the lower limb equations (nine out of 14 equations (64%)) (Table 7). Moreover, both FORDISC equations yielding the Bayes factors greater than 10 were from the upper limb equations (“Radius” and “Humerus+Radius” equations) (Table 7). As the primary purpose of this study is to report the comparative performance of the methods/equations, exploring the reason for the different performance among equations is beyond the scope of this study and needs to be a topic for the future research.

Lastly, exclusion of the Black male equations from the analysis due to insufficient sample size is another limitation of this study. Thus, a validation test of the Black male equations should be another topic for the future research with additional data.
